# Reproductive Toxicity and Life History Study of Silver Nanoparticle Effect, Uptake and Transport in *Arabidopsis thaliana*

**DOI:** 10.3390/nano4020301

**Published:** 2014-04-22

**Authors:** Jane Geisler-Lee, Marjorie Brooks, Jacob R. Gerfen, Qiang Wang, Christin Fotis, Anthony Sparer, Xingmao Ma, R. Howard Berg, Matt Geisler

**Affiliations:** 1 Department of Plant Biology, Southern Illinois University Carbondale, Carbondale, IL 62901, USA; E-Mails: jgerfenagedu@siu.edu (J.R.G.); cfotis117@gmail.com (C.F.); tjsparer@siu.edu (A.S.); 2 Department of Zoology, Southern Illinois University Carbondale, Carbondale, IL 62901, USA; E-Mail: mlbrooks@siu.edu; 3 Department of Civil and Environmental Engineering, Southern Illinois University Carbondale, Carbondale, IL 62901, USA; E-Mails: denniswang@siu.edu (Q.W.); ma@engr.siu.edu (X.M.); 4 Integrated Microscopy Facility, The Donald Danforth Plant Science Center, St. Louis, MO 63132, USA; E-Mail: rhberg@danforthcenter.org

**Keywords:** *Arabidopsis thaliana*, silver nanoparticles, life history traits, transport

## Abstract

Concerns about nanotechnology have prompted studies on how the release of these engineered nanoparticles impact our environment. Herein, the impact of 20 nm silver nanoparticles (AgNPs) on the life history traits of *Arabidopsis thaliana* was studied in both above- and below-ground parts, at macroscopic and microscopic scales. Both gross phenotypes (in contrast to microscopic phenotypes) and routes of transport and accumulation were investigated from roots to shoots. Wild type Arabidopsis growing in soil, regularly irrigated with 75 μg/L of AgNPs, did not show any obvious morphological change. However, their vegetative development was prolonged by two to three days and their reproductive growth shortened by three to four days. In addition, the germination rates of offspring decreased drastically over three generations. These findings confirmed that AgNPs induce abiotic stress and cause reproductive toxicity in Arabidopsis. To trace transport of AgNPs, this study also included an Arabidopsis reporter line genetically transformed with a green fluorescent protein and grown in an optical transparent medium with 75 μg/L AgNPs. AgNPs followed three routes: (1) At seven days after planting (DAP) at S1.0 (stages defined by Boyes *et al.* 2001 [41]), AgNPs attached to the surface of primary roots and then entered their root tips; (2) At 14 DAP at S1.04, as primary roots grew longer, AgNPs gradually moved into roots and entered new lateral root primordia and root hairs; (3) At 17 DAP at S1.06 when the Arabidopsis root system had developed multiple lateral roots, AgNPs were present in vascular tissue and throughout the whole plant from root to shoot. In some cases, if cotyledons of the Arabidopsis seedlings were immersed in melted transparent medium, then AgNPs were taken up by and accumulated in stomatal guard cells. These findings in Arabidopsis are the first to document specific routes and rates of AgNP uptake *in vivo* and *in situ*.

## 1. Introduction

The proliferation and release into the environment of engineered 1–100 nm diameter nanoparticles [1] worldwide raises important ecological and human health concerns. Aquatic and terrestrial microbes, plants and animals are profoundly affected by nanoparticles [2,3,4,5]. Moreover, engineered nanoparticles have been detected in wastewater, indicating potential human exposure and health concerns [6,7,8,9,10]. The current and future environmental and health problems stemming from nanotechnology should be understood before these problems become too serious to be resolved [11,12,13,14,15,16]. Among all engineered nanoparticles, silver nanoparticles (AgNPs) are the most widely used; approximately 800 megatons (1 × 10^6^ tons) of global use per year, among many different industries, due to their unique antimicrobial properties [14,17]. Unfortunately, AgNPs wash out of products, which release them into the environment and the human food chain [18,19].

Studies on silver nanotoxicity (toxicity due to AgNPs) in plants have been controversial [16,20,21,22,23], centered on whether the cause of toxicity is either the nano-size and shape of the particles or their release as ionic Ag^+^. All studies, however, agree that silver nanotoxicity is positively concentration-dependent and negatively size-dependent [20,23,24,25]. AgNPs are reported to accumulate progressively in the *Arabidopsis thaliana* root tip, from border cells, to root cap, columella, and columella initials of the root meristem [20]. In the first 1–2 mm of the root tip, AgNPs were transported via the intercellular spaces and aggregated at plasmodesmata (connections between two plant cells). The diameter of plasmodesmata is approximately 50–60 nm [26,27]. Research using 45 nm fluorescent NaYF4:Yb,Er nanoparticles in the larger (than Arabidopsis) aerial roots of the moth orchid plant (*Phalaenopsis* spp.), showed nanoparticles were transported from root to shoot through xylem within five days [28]. Although the same study also found that Arabidopsis leaves contained NaYF4:Yb,Er nanoparticles, their transport routes and speed were not described. Ma *et al.* [4] proposed that vascular tissues might play a significant role in the long-distance bulk transport of nanoparticles. Another study reported that CuO nanoparticles in maize (*Zea mays*) were transported upward by xylem and downward by phloem using transmission electron microscopy [29].

Root tip accumulation of nanoparticles is important because the tip contains stem cells (*i.e.*, undifferentiated cells able to divide into many tissue types) such as root meristem and collumela initials that are needed for root growth and production of the root cap. Stem cells were early targets of silver nanotoxicity in mammalian cells [30] and nanoparticles were genotoxic (damage on DNAs) [31,32,33]. Fortunately, plants can somewhat mitigate genotoxicity by producing lateral roots (derived from the root pericycle) or adventitious roots (derived from the cortex of the hypocotyl) if the primary roots are damaged [34]. Flow of hormone auxin from shoot to root can also trigger early lateral root formation [35]. This leads to the hypothesis for Arabidopsis that like primary root apical meristem, the lateral root primordia are vulnerable to silver nanotoxicity if unbound nanoparticles occurred in their growth media. If the newly formed lateral root primoridia are not affected, then their resistance could be a coping mechanism against silver nanotoxicity, protecting the progenitor cells in the pericycle or cortex of the developing lateral root.

Clearly, if some of these cells survive, then increased lateral root formation could develop to compensate for the loss of primary root growth; continuing to absorb the water and nutrients that sustain a plant’s overall growth. Lateral root formation depends on the mechanical properties of its overlapping tissues [36]; thus, if some primordial cells are damaged, the entire primordium may be aborted. Geisler-Lee *et al*. [34] also showed that overexpression of cell plate-associated dynamin-related proteins, phragmoplastin, arrested the primary root growth. Numerous new lateral and adventitious roots were then initiated, but these aborted also. In addition, the few surviving cells in the original primary root apical meristem terminally differentiated into vascular tissues. Thus, if silver nanotoxicity could persist in damaging stem cells of lateral root primordia, e.g., by regular exposure to AgNPs, these too would abort in early stages of development and few lateral roots would develop and grow. Field studies have shown plants could survive nanotoxicty but nanoparticles caused adverse ecosystem responses [3,18].

Contemporary nanotoxicity studies in plants, including crop species, have focused on acute effects (within hours and/or days) and hydroponic growth [37,38,39]. These hydroponic growth assays and acute stress studies can evaluate acute and immediate nanotoxicity effects in plants, but may miss long term chronic effects of low dose exposure, which might have different targets and pathology. In contrast, a life history trait study in soil will help answer how plants behave and survive when they are chronically exposed to nanoparticles. Plants may show strong resilience to the weeks or months of chronic nanotoxicity. These plants may be able to complete their life cycle by employing developmental or adaptive changes (such as increased lateral root emergence). If so, the plants’ phenotype may change when experiencing chronic silver nanotoxicity. To address the questions of long term exposure to nanoparticles, we present a comprehensive study of the life history traits of AgNP-treated *A. thaliana*, including AgNP transport and accumulation in both below- and above-ground parts.

## 2. Materials and Methods

### 2.1. Chemicals

Citrate-stabilized silver particles (20 nm, 7.0 × 10^10^ particles/mL) were purchased from Ted Pella Inc. (Redding, CA, USA) and used immediately upon receipt. The AgNPs from 2 different batches of the same product (and catalog number) from this supplier were previously characterized and were consistent from batch to batch [20]. Although long term storage and changes in manufacturing process can significantly alter particle size distributions, since this supplier delivered relatively consistent AgNP batches, and there was no long term storage after arrival, further characterization of individual batches was deemed unnecessary. The monodisperse AgNPs could aggregate into much larger sizes after mixing with quarter-strength Hoagland solution [40] and become polydisperse [20]. However the aggregation, adhesion, and dispersion of AgNPs when within the soil media was not tested.

### 2.2. Seeds and Germination in Soil

*Arabidopsis thaliana* Columbia ecotype seeds were purchased from Lehle Seeds (Round Rock, TX, USA). Seeds were pretreated as previously described [20], *i.e.*, with 15% Clorox (common household bleach; NaOCl) for 10 min to remove any microbial contamination and were then rinsed thoroughly with sterile reverse osmosis water (herein, ddH_2_O) three times. Seeds were sown directly into soil afterwards. Three seeds were placed per pot. Seeds germinated and grew in 100 mm × 100 mm × 125 mm pots with Fafard^R^ 4M Mix (Conrad Fafard Inc., manufacturer No. # 8063028, Agawam, MA, USA) potting soil. Each pot initially contained a total weight of 112 g of dry soil (prior to watering). Freshly produced Arabidopsis seeds (for about a year after seed set) need a cold exposure to overcome dormancy; a process known as stratification. This was done at 4 °C in the dark for 4 days, in soil-filled pots (in a tray, dimensions = 10" × 20") covered with a 7" vented humidity dome (Mondi Mini Greenhouse 7" Propagation Dome, dimensions = 10" × 20" × 7") to maintain moisture for seed germination and later transferred to a growth chamber with a long day light cycle [20]. To prevent seedling death, seedlings were slowly acclimatized from being under the humid dome to directly exposure to light from 14 to 21 days after planting (DAP) (Table S1).

### 2.3. Life History Trait Study

Seventy-five and 300 μg/L of AgNP suspension in ddH_2_O was used for irrigation. Plants were irrigated with either ddH_2_O, AgNPs, or an equivalent dosage of AgNO_3_ based on the amount of silver ions the AgNPs at that concentration were shown to generate in solution [20]. The two dosages of AgNO_3_ were 4.25 and 17 μg/L. The irrigation was done twice a week and the overall volume irrigated over the life history was 960 mL per plant (see TTable S1 for the dosages and irrigation schedules). Developmental stages were characterized based on the established scales by Boyes *et al.* for *A. thaliana* [41]. Digital images were taken through Leica FireCam version 3.4 for Mac by a Leica EC3 digital camera connected with a Leica dissection microscope and by Cannon PowerShot A620 digital camera. Images were taken every 3 days on shoots (aboveground parts) until S1.10 stage, and then a Ward’s forensics ruler (#CSM8X8) was used for rosette diameter measurements. Digital images of early rosette leaves (prior to S1.10) were analyzed quantitatively with the ImageJ software (National Institutes of Health, Bethesda, MD, USA). The entire life history study was replicated 3 times.

Major growth stages were as followed. S0: imbibition; S0.70: hypocotyl and cotyledon emergence; S1.00: two cotyledons fully opened; S3.20: rosette is 20% of final size; S5.10: first flower bud visible; S6.00: first flower open; S6.90: flowering complete; S9.0: senescence [41].

### 2.4. Germination Assay

One hundred seeds per treatment were germinated on filter paper (Fisherbrand Filter Paper; Cat. No: 09-795B; Quantitative P8; 7.0 cm diameter in size) in a Petri Dish (Kimble Kimax 23060-10020 Borosilicate Glass 100 mm × 20 mm) with 2 mL of either ddH_2_O or 75 μg/L AgNPs (in ddH_2_O). Seed germination was counted at 10 DAP. Seeds produced by healthy unexposed plants (exposed seed generation 0 or E0) were growing in exposed soil until seed harvest. The seeds from this harvest (E1) were themselves grown on exposed soil until seed harvest to produce the E2 seed generation, and so on for the E3 seed generation. To test reproductive toxicity, germination rates of all 4 generations of seeds (E0–E3) were compared. Each treatment had triplicate germination tests and the entire experiment was repeated three times.

In brief, E0–E3 generations were defined as followed (also see Figure S1):

E0: generation: seeds were harvested from non-exposed plants;

E1: seeds were harvested from the plants germinated from E0 seeds, either irrigated with water or AgNPs;

E2: seeds were harvested from the plants germinated from E1 seeds, either irrigated with water or AgNPs;

E3: seeds were harvested from the plants germinated from E2 seeds. Different letters indicate significantly different.

### 2.5. Determination of Silver Content in Soil and Plant Tissue—Sample Preparation

At S6.0 (first flower open) and S9.0 (first silique shattered), shoots and roots were harvested separately, quick dried in liquid N_2_ and stored in −80 °C until use. After tissue removal, soil was collected in a Ziploc bag and stored in 4 °C until analysis. Samples (soil, roots or shoots) were freeze-dried for 12–24 h until they achieved a consistent dry weight (dw). Approximately 0.5 g of dried sample was microwave digested (MARSXpress digestor, CEM Corp., Matthews, NC, USA) in trace metal grade nitric acid (TMG-HNO_3_) (Fisherbrand, Fisher Scientific, Pittsburg, PA, USA). The samples were then diluted to a concentration of 5% HNO_3_ with ultrapure 18 megaOhm (MΩ) water. Concentrations of Ag (μg of Ag per gram dw of sample) were determined via graphite furnace atomic absorption spectrophotometry (GFAAS) according to the U.S. Standard Methods [42]. To remove any undigested inorganic residue, samples were filtered through exhaustively pre-rinsed 0.7 µm glass fiber filters (Whatman brand GF/F, GE Healthcare Life Sciences, Piscataway, NJ, USA). All vessels for digestion and analysis of samples were acid-washed. Method blanks showed no contamination of samples from the filters or from acid-washed vessels.

Percent organic material of soil was determined from the ash free dry mass (AFDM) after combusting approximately 0.5 g dry weight of soil in a muffle furnace at 500 °C for one hour. Percent organic material was determined by subtracting AFDM from the dry mass.

### 2.6. Determination of Silver Content in Soil and Plant Tissue—Quality Assurance/Quality Control

Quality assurance protocols included internal and external quality checks included in all analytical runs. Quality checks, included every 10 samples, consisted of calibration blanks (CB), calibration verifications (CV), sample duplicates, and sample spikes. The CB was the running matrix (5% TMG-HNO_3_ in 18 MΩ water). The CV was the 25 μg/L standard analyzed as a sample. Sample duplicates consisted of a separate replicate analysis of the same sample. Sample spikes consisted of a mix of half sample and half running standard (*v*/*v*), with expected concentrations of 12.5 μg/L + ½ μg/L of the mean value of the unamended sample. In addition, at the beginning of each analytical run, we analyzed an external quality check (EQC). The EQC consists of an externally-prepared certified reference material at 20 μg/L (EQC; Fluka^®^ EQC Reference Standard for GFAAS and ICP-MS, SigmaAldrich Corp., St. Louis, MO, USA).

### 2.7. Soil Nutrient Analysis

At S6.0 ad S9.0, after above- and below-ground tissues were harvested, their soil was subjected to nutrient analysis. The extraction method for soil followed Keeney and Nelson [43]. Ten grams of soil were weighed into a 125 mL Erlenmeyer flask, then was added 50 mL of freshly made 2 N KCl and shaken at 200 rpm for one hour. Afterwards, the mixture was filtered through 0.4 μm filter. The filtrate was segmented by flow analysis at an OI Analytical Flow Solution IV (OI Analytical Corp., College Station, TX, USA) for NO_3_^−^ and NO_2_^−^. The nitrate test followed U.S. Environmental Protection Agency method 353.2 [44]. Nitrate is reduced to nitrite by cadmium metal.

### 2.8. Seeds and Seed Germination in Gel Media

One Arabidopsis *in vivo* endoplasmic reticulum (ER) localized green fluorescent protein marker line (herein, ER::GFP) [45] from Arabidopsis Biological Research Center was used. ER::GFP lines are in the Columbia ecotype background that their phenotypes could be comparable to those of the Columbia ecotype. ER::GFP seeds were pretreated as described. Seeds were sown directly afterwards. Seeds germinated and grew in a Magenta™ box (GA-7, dimensions = 76.2 mm × 76.2 mm × 101.6 mm) with quarter (1/4) strength Hoagland media [40] with 0.3% (*w*/*v*) Carrageenan (Product ID: C257, PhytoTechnology Laboratories; Kappa-Type Carrageenan) under sterile conditions. Hoagland solution was from Connecticut Valley Biological Supply Company (Model: A 2022; Southampton, MA, USA). Carrageenan is optical transparent [46] and suitable for digital imaging; it was used to physically support seedling/plant growth. A mixture of one-quarter strength of Hoagland solution with 0.3% (*w*/*v*) of Carrageenan was sterilized by autoclave first. When the sterilized mixture cooled down to 60 °C, 20 nm AgNPs were added to a concentration of 75 μg/L. Each box contains a total volume of 150 mL of gel media with or without 75 μg/L of 20 nm AgNPs. Once the mixture was solidified, nine seeds were sown on the surface of the gel media. After stratification at 4 °C for 4 days, gel boxes were transferred to a growth chamber with a long day light cycles as described above.

### 2.9. Detection of Silver in Plant Tissues by Confocal Microscopy

The previous method [20] with modification in two channels of emission bandpass was used in this study: 500–550 nm excitation for GFP and 480–520 nm for AgNPs. 488 nm excitation (for AgNPs) from an Argon gas laser was used. Artificial green color was assigned to present GFP florescence while red color to silver reflection signals. Seedlings of 7, 14, 17 DAP were gently removed from their box in a sterile hood prior to be hand sectioned and then observed under an LSM510 confocal microscope as previously described [20]. The three dates were tested to observe when AgNPs entered the vascular system.

### 2.10. Detection of Silver Element on Surface of Roots by Scanning Electron Microscopy (SEM) and Energy Dispersive X-Ray Spectroscopy (EDS)

The Hitachi Analytical Tabletop Microscope/Benchtop SEM TM3030 (Hitachi High Technologies America, Inc., Schaumburg, IL, USA) and the Bruker Quantax 70 Energy-Dispersive X-Ray Spectroscopy (Bruker Corporation, Billerica, MA, USA) were used to view AgNP accumulation in the roots of 14 DAP and verify the presence of elemental Ag, respectively. Several whole seedlings from gel boxes were carefully excised from the gel medium and subjected to be viewed and analyzed by a Hitachi TM3030 (Hitachi High Technologies America, Inc., Schaumburg, IL, USA).

### 2.11. Data Analysis

The data were analyzed and plotted by Sigma Plot version XI. Students’ *t*-test was performed between groups, and one-way ANOVA used in bar graphs to distinguish significantly different sample averages. *p* value <0.05 is deemed significant, unless specified otherwise.

## 3. Results

### 3.1. Chronic Exposure Resulted in Prolonged Vegetative and Shortened Reproductive Growth

**Figure 1:**
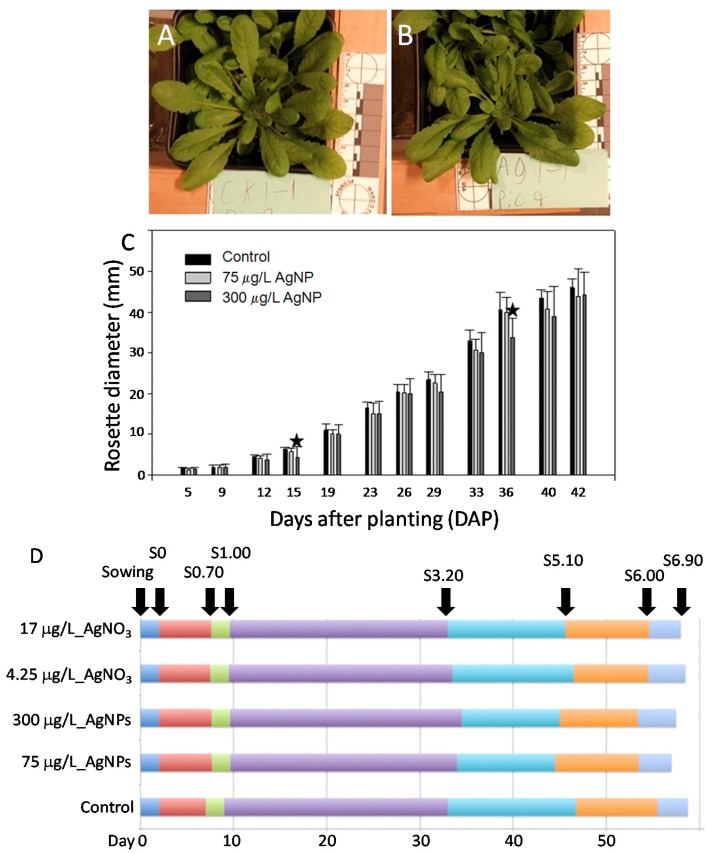
Study of life history traits of Arabidopsis plants irrigated with ddH_2_O or with AgNPs. (**A**,**B**) Morphology of Arabidopsis rosette leaves growing in potted soil. (**A**) Control plants irrigated with ddH_2_O; (**B**) treated plants with 75 μg/L of 20 nm AgNPs. (**A**) and (**B**) were at S3.50. (**A**) and (**B**) showed no distinct and visual differences. (**C**) Growth of aboveground vegetative parts of control and AgNP-treated within 42 days after planting (DAP). (**D**) Chronological progression of control, AgNPs-treated (both 75 and 300 μg/L) and AgNO_3_-treated (4.25 and 17 μg/L) plants from sowing to S6.90.

Within 42 DAP at S3.50 stage, *i.e.*, its rosette is 50% of final size, there was no distinct morphological difference in control and 75 μg/L AgNP treated plants (Figure 1A,B). However, there were significant differences in control and 300 μg/L AgNP treated plants at 15 and 36 DAP (Figure 1C). Aboveground parts include cotyledon and true leaves. Significant differences (*; *p* < 0.05) between control and 300 μg/L of AgNP-treated plants showed at 15 DAP (S1.00) and 36 DAP (S3.20). These two dates coincided with developmental stages S1.00 (two cotyledons are fully open) and S3.20 (rosette is 20% of final size), respectively. These two dates also mark the beginnings of the principal growth stage 1 for leaf development and the principal growth stage 3 for rosette growth, respectively. These implied that at these two developmental stages, Arabidopsis plants were susceptible to a high concentration 300 μg/L of AgNPs to show significant differences in rosette growth. In terms of the whole life history, from 0 DAP to S6.90 (flowering complete), AgNP-treated plants had three features: a longer principal growth stage 1, a shorter rosette growth from S3.20 to S5.10 (inflorescence emergence) and a shorter reproductive stage (including inflorescence and flower production) from S5.10 to S6.90 (3–4 days shorter) (Figure 1D). The principal growth stage 1 was 2–3 days longer; the reproductive stage was 3–4 days shorter. However, AgNO_3_ treatment, although toxic at both 4.25 and 17 μg/L [20], did not alter life cycle stage progression compared to controls.

Two concentrations were chosen, the lowest (75 μg/L) and highest (300 μg/L), and the smallest size (20 nm) of three (20, 40, 80 nm) was chosen based upon results of a previous study [20]. Although these were insufficient to create a dose response or size effect analysis, these concentrations were sufficient to determine the presence and nature of long term effects of AgNP exposure on plant growth phenotype and reproductive fitness. The 20 nm size of particle was also shown to have the strongest effect of all AgNP sizes in the previous study [20].

### 3.2. Reproductive Toxicity

**Figure 2:**
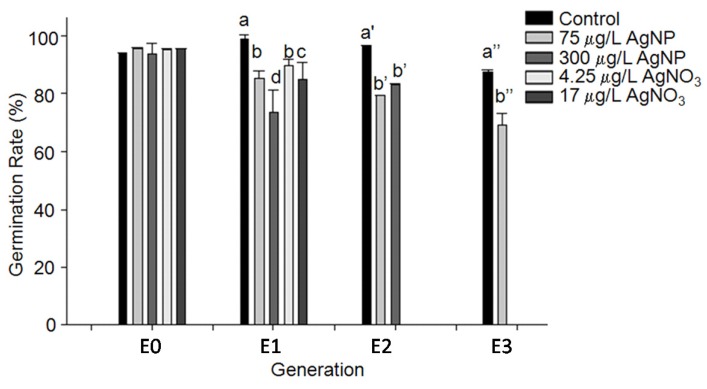
Reproductive nanotoxicity of AgNP and AgNO_3_ in Arabidopsis. This showed 20 nm AgNPs affected seed germination; it also showed stronger toxicity of AgNPs than that of AgNO_3_ on germination in E1 generation. Different letters indicate significantly different.

Chronic exposure to AgNPs resulted in no significant difference in reproductive tissue development, e.g., inflorescence height, flower number, silique number, and seed weight (Figure S2). However, seed germination rates decreased from the initially exposed E0 generation (see the Section 2 for the definitions) through E1 to E3 generations (Figure 2). In E1 generation, the seeds harvested from 300 μg/L of AgNPs treated plants showed the lowest germination rate among 5 treatments: control, 75 *vs.* 300 μg/L of AgNPs and their releasing Ag^+^ concentrations (*i.e.*, 4.25 *vs.* 17 μg/L, respectively). In E2 generation, the seeds from both 75 and 300 μg/L of AgNPs had similar toxic effect on seed germination. By the E3 generation, the germination rate of the seeds harvested from the plants irrigated with 75 μg/L of AgNPs decreased to approximate 70% of E0 seeds.

### 3.3. Transport of Ag from Root to Shoot

**Figure 3:**
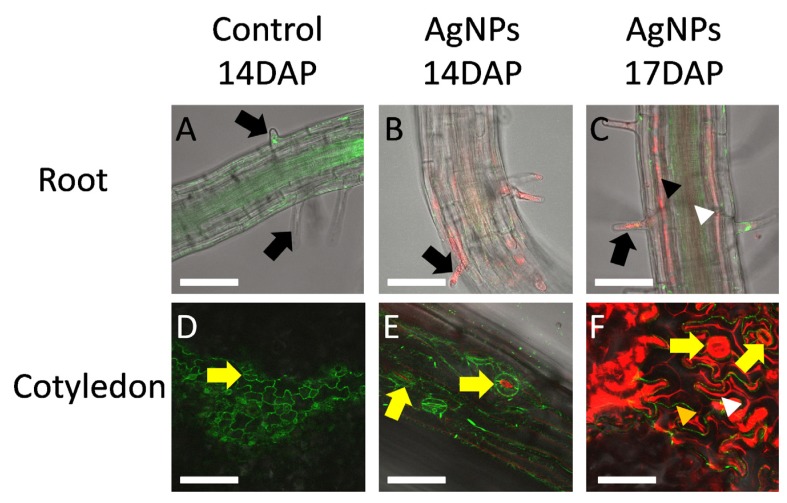
Transport of AgNPs in Arabidopsis ER::GFP plants. Seedlings of 14 (**B**,**E**) and 17 (**A**,**C**,**D**,**F**) days after planting (DAP) were examined under a Zeiss LSM 510 confocal microscope. (**A,C**) came from root sections of maturation region; (**D**–**F**) came from cotyledon. (**A**) and (**D**) are control; (**B**,**C**) and (**E**,**F**) are AgNPs-treated. Green color was intrinsic GFP; red color was Ag^0^ light scattering. At 14 DAP, AgNPs accumulated mainly in root hair cells and surface of roots (**B**). By 17 DAP, AgNPs already entered vascular tissue, both phloem (white arrowhead) and xylem (black arrowhead), of the roots and could be bulk transported through vascular tissue. Upon germination, some condensed media might have touched cotyledons. At 14 DAP, AgNPs could be observed in the pores of stomata (yellow arrows in **E**). By 17 DAP, not only the pores of stomata but also the stomata themselves (yellow arrows in **F**) showed AgNP accumulation. The uneven surface of pavement cells [56] showed AgNP accumulated on the grooves (white arrowhead) between pavement cells (orange arrow). Scale bar = 0.2 μm.

In the current study, Arabidopsis seeds were germinated in a gel medium and their seedlings were observed to track how AgNPs were transported in roots and from roots to whole plants. In addition to AgNP accumulation on root surface and root tips [20] (Figure S3A), AgNPs were found in root hair cells (Figure 3B, Figure S4B), Figure S5) at 14 DAP. By 17 DAP, AgNPs had entered vascular tissue, both xylem and phloem (Figure 3C, Figure S4C) and could be bulk-transported throughout the whole plant. Some lateral roots appeared undamaged but others were obviously affected (Figure S6A–C). Cotyledons were also observed to track possible alternative routes of AgNP entry. Cotyledons emerged in direct contact of their growth medium surface that AgNPs could enter cotyledons through the pores of stomata (Figure 3E, Figure S4F) at 14 DAP. By 17 DAP, AgNPs already heavily accumulated in stomata and the cell wall grooves of adjacent pavement cells (Figure 3F, Figure S4F).

In addition to using reflected light signals in confocal microscopy, scanning electron microscopy (SEM) and energy dispersive X-ray spectroscopy (EDS) were performed to view the presence of Ag in root hair cells (Figure S5). Thus, two parallel microscopic studies, confocal microscopy and SEM, verified accumulation of AgNPs in root hair cells and root tips (Figures S5 and S7).

### 3.4. Silver Accumulation in Plant Tissues and Remainder in Soil

**Figure 4:**
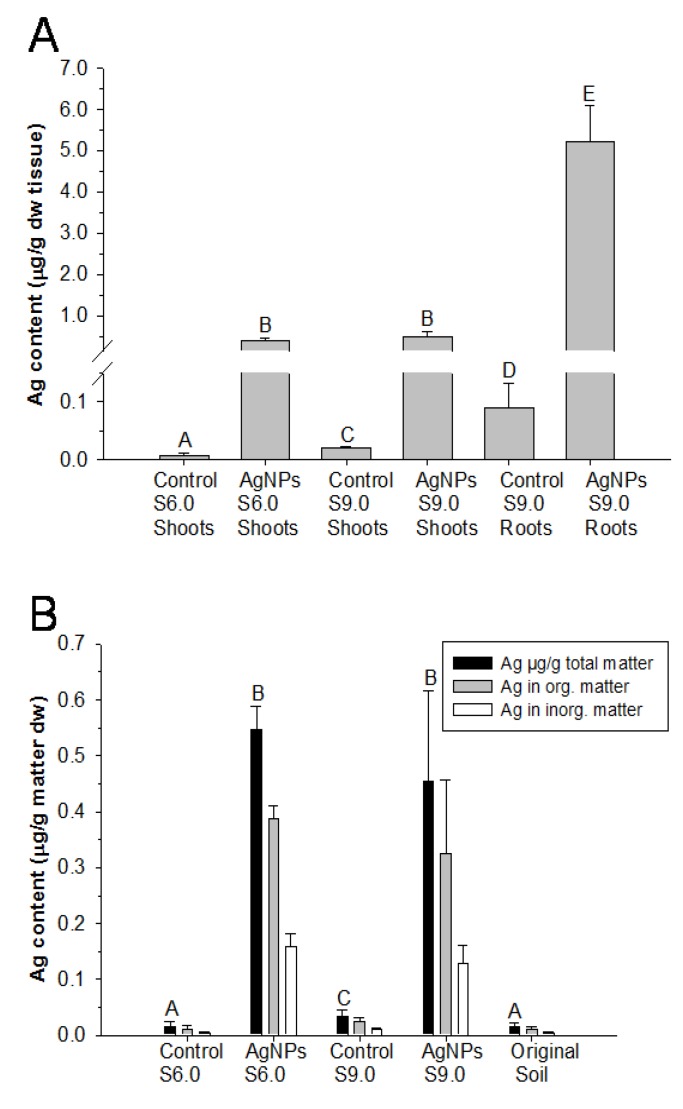
Measurements of silver contents in plant tissues and soil matter. (**A**) Silver accumulation in aboveground parts (shoots) and belowground parts (roots) at the growth stages of S6.0 and S9.0. (**B**) Silver accumulation in AgNP exposed soil after plants were harvested at S6.0 and S9.0 in terms of μg per g of dry weight of soil sediment with μg of AgNP relative to the fraction of organic and inorganic matter. The presence of AgNPs in control samples might have been due to human errors, *i.e.*, misirrigation of AgNP suspension instead of ddH_2_O. See the Materials and Methods for the definition of dry weight, organic and inorganic matter. Different letters indicate significantly different soil or tissue concentrations (*t*-tests, *p* = 0.05). Abbreviations: dw, dry weight; inorg, inorganic; org, organic; original soil, potting soil from Fafard^R^ 4M Mix.

Two principal growth stages, S6.0 and S9.0, were used to measure the accumulation of Ag in aboveground (e.g., shoots) and belowground (*i.e.*, roots) tissues. By S9.0, roots accumulated 10 times more Ag than shoots did; however, there was no difference of Ag accumulation in aboveground tissue at both S6.0 and S9.0 stages (Figure 4A).

The soil from these two stages was also subjected to Ag content analysis. This showed significant differences in total Ag in both the S6.0 and S9.0 soil samples relative to controls and original potting soil from Fafard^R^ 4M Mix (Figure 4B); however, there was no difference in total Ag in both the S6.0 and S9.0 soil samples. The significant, but small absolute difference between the S6.0 and S9.0 control soils was likely due to a single accidental addition of AgNP suspension to the S9.0 control soil at some point throughout the duration of the experiments (Table S1).

### 3.5. Consumption of Soil Inorganic Nitrogen Nutrient

**Figure 5:**
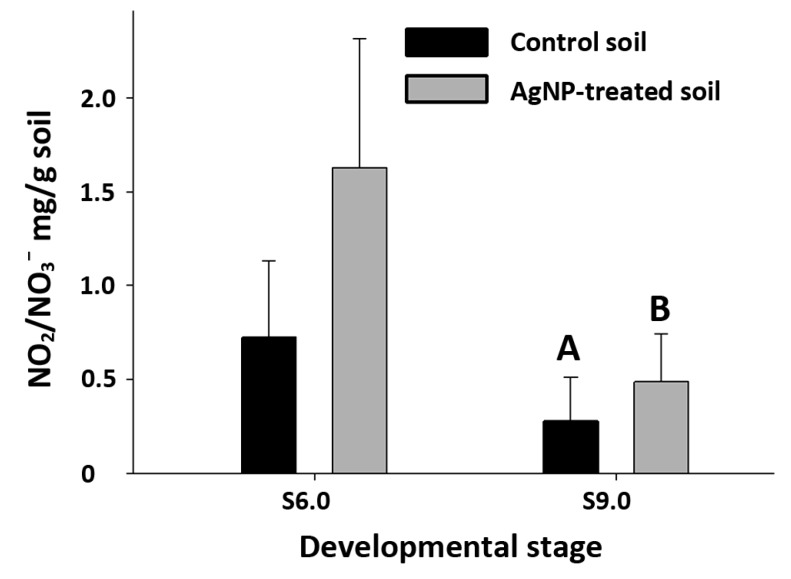
Inorganic nitrogen nutrients in soil. Inorganic nitrate/nitrite contents of soil were measured after plant tissues were harvested at S6.0 and S9.0. At S6.0, there was no significant difference between control and treated soil. However by S9.0, more nitrate/nitrite remained in the AgNP-treated soil than control soil.

With the presence of AgNPs/Ag in the soil, plants might have difficulty in absorbing nutrients, taking inorganic nitrogen (both nitrate and nitrite) for example (Figure 5). But this difference did not occur until later in the plant life history. At the S6.0, there was no difference of inorganic nitrogen nutrient in both control and AgNP-treated soil. However, at the S9.0, there was a difference between two groups (Figure 5). Reproductive growth might have demanded more nutrient uptake and thus depleted the soil.

## 4. Discussion and Conclusions

### 4.1. Silver Nanoparticles Are a Newly Man-Made Abiotic Stressor that Alters the Plant Life Cycle and Subsequent Generations

High concentrations of AgNP treatments can cause temporary development arrest resulting in an extended vegetative phase in Arabidopsis. Although such a lower dosage as 75 μg/L of AgNP treatment did not cause any significant differences in aboveground (rosette) growth, it still had the effect of prolonging vegetative development. This suggests that there is some obstacle Arabidopsis plants encountered during their vegetative development between stages S1.0 to S3.2. Once the obstacle could be overcome and vegetative development could proceed further. We speculate that this arrest is likely coinciding with the loss of belowground function of the primary root meristem and that the delay is due to redirection of resources to lateral root formation. There may also be an effect at the dosage of the 75 μg/L of AgNP treatment on the aboveground morphological phenotype that might be too subtle during our gross biometric measurements (rosette diameter) to detect. After the temporary developmental arrest occurred and was overcome, AgNP-treated plants seemed to proceed to a faster and shorter reproductive development perhaps in order make up for lost time, or to avoid the stress by setting seed. This phenomenon of early flowering and seed filling has been reported in Arabidopsis and other plants affected by other stressors, both biotic or abiotic [47,48,49]. In addition to longer vegetative and shorter reproductive development, Arabidopsis plants also suffer gradual degenerative seed viability with a decreasing germination rate in successive generations. There was no difference in seed germination among E0 seeds, of which result was similar to that in literature [50]; however, as nanoparticle exposed (E1) generation and following generations (E2,E3) had increasingly diminished germination rates. This is the first such reporting in plants. However in animal research, generation level effects of nanoparticle exposure in the water flea *Daphnia magna* study showed TiO_2_ nanoparticles increase sensitivity in the next generation [2].

### 4.2. Accumulation and Long-Distance Transport of Silver Nanoparticles in Arabidopsis Plants

Confocal microscopy was used to present the differential and distinctive accumulation patterns of both AgNPs and Ag^+^ (from AgNO_3_) [20]. Only AgNPs and Ag^0^, but not Ag^+^, could be observed due to light reflection of silver element (Ag^0^). AgNPs caused a gradual accumulation in a time sequence in the root tip and elongation region during four weeks of hydroponic growth. A root includes three regions, root tip, elongation region and maturation region. Root tips include stem cells and differentiated root cap cells; elongation region includes divided cells which undergo elongation; maturation region includes differentiated cells with specific functions. In addition to observing Arabidopsis roots over a wider range of growth stages, we observed and reported the maturation region of Arabidopsis roots. AgNPs entered root hair cells at 14 DAP and then vascular tissue, both phloem and xylem, by 17 DAP. Once AgNPs were in the vascular tissues, they could be transported throughout the whole plant, *i.e.*, long-distance transport. This route of transport confirmed the proposed scheme by Ma *et al.* [4]. This also supported the fact of Ag accumulation in Arabidopsis shoots when they were treated with such a sublethal dosage as 1 mg/L of 5 nm of AgNPs [51]. An amount of 1 mg/L is more than 13 times more of 75 μg/L used in this study; 5 nm is ¼ time of 20 nm in size. Early accumulation in cotyledons was observed. At 14 DAP, few AgNPs entered the pore (opening) of a stoma. But mere three days later, heavy accumulation of AgNPs could be observed throughout the surface of cotyledon, mainly pavement cells and stomata. This study on long-distance transport and lifelong tolerance to AgNPs in Arabidopsis agreed with a study in soybean *Glycine max* when amended with either ZnO or CeO_2_ nanoparticles [52]. In the study of ZnO or CeO_2_, transmission electron microscopy (TEM) was performed to observe the locations of nanoparticles in fixed cells and tissue. However in the current study, confocal microscopy was performed to observe the locations of AgNPs *in situ* and *in vivo*.

### 4.3. Stem Cells and Silver Nanotoxicity

It has been reported that damage to stem cells (*i.e.*, undifferentiated meristematic cells which can divide) like columella initials in root tips was the first phenomenon to be observed [20,29,53,54]. Not only primary root tips but also lateral root primordia (Figure S6A–C) were affected by AgNPs. Once the Arabidopsis primary root apical meristem was destroyed by AgNPs, lateral root primordia were initiated. If some of lateral root primordia survived silver nanotoxicity, they would continue to divide, elongate and differentiate, and eventually grow into fully functional lateral roots which would take over for the function of the primary root. Subsequently these lateral roots would support the growth and development of Arabidopsis plants against exposure of AgNPs. Perhaps the prolonged vegetative growth between S1.00 and S3.20 was due to the time delay for enough surviving lateral roots to accumulate and produce functional roots for water and nutrient absorption. Future studies will examine overall root surface area and volume as a possible explanation for the delay in growth phase.

### 4.4. AgNPs May Affect Arabidopsis Plants’ Nutrient Uptake from Soil

Silver nanotoxicity has been considered to be due to the released Ag^+^ from AgNPs with minor/partial contribution of the nano-size of AgNPs. There is little immediate change in differential gene expression specific to acute exposures to AgNPs [21]. However, we observed a change in inorganic N-nutrients (e.g., nitrate and nitrite) absorption by chronic AgNP-treated Arabidopsis plants at S9.0. This probably would have a corresponding effect on expression of nutrient uptake gene pathways. Unlike Ag^+^ treatments, AgNPs were found aggregated at plasmodesmata and in cell wall [20]. This suggests there would be wall leakage and blocking of intercellular communication due to the mechanical presence of nano-sized particulates as AgNPs at these sites. Our results could also imply that AgNPs or their released Ag^+^ affect the function of nutrient transporter proteins. These proteins help lessen clogging of plasmodesmata and regulate intercellular transport [55]. Unfortunately the disentanglement of causality of silver nanotoxicity (particle or ion) is, so far, still somewhat murky. However, this study clearly shows that there are additional toxic effects for AgNPs (*vs.* Ag^+^ ions) over the life cycle and subsequent generations of Arabidopsis plants during chronic exposure.

## Supplementary Materials

Supplementary File 1
