# Supplementary material for: Reproductive Toxicity and Life History Study of Silver Nanoparticle Effect, Uptake and Transport in Arabidopsis thaliana
Source: Nanomaterials (Basel). 2014 Apr 22;4(2):301–18. doi: 10.3390/nano4020301 (PMC5304678; doi:10.3390/nano4020301)
Supplement: Supplementary File 1 [file nanomaterials-04-00301-s001.pdf]

Supplementary Information

Table S1. The dosages of irrigation per plant over the growth period. The total volume was 960 mL.

Days after planting (DAP)	Volume (mL)	Event(s)
0	0	Sowing seeds
4	0	Transferred from 4 °C to 20 °C
14	3	Dome 1/4 opened
18	3	Dome 1/2 opened
21	3	Dome completely open
25	3	
28	3	
32	3	
35	9	
39	9	
42	9	
46	45	
49	45	
53	45	
56	45	
61	45	
63	45	
67	45	
70	60	
74	60	
77	60	
81	90	
84	90	
88	120	
91	120	
95	0	Tissue harvest & soil collection

Figure S1. Illustration of E0–E3 generations.

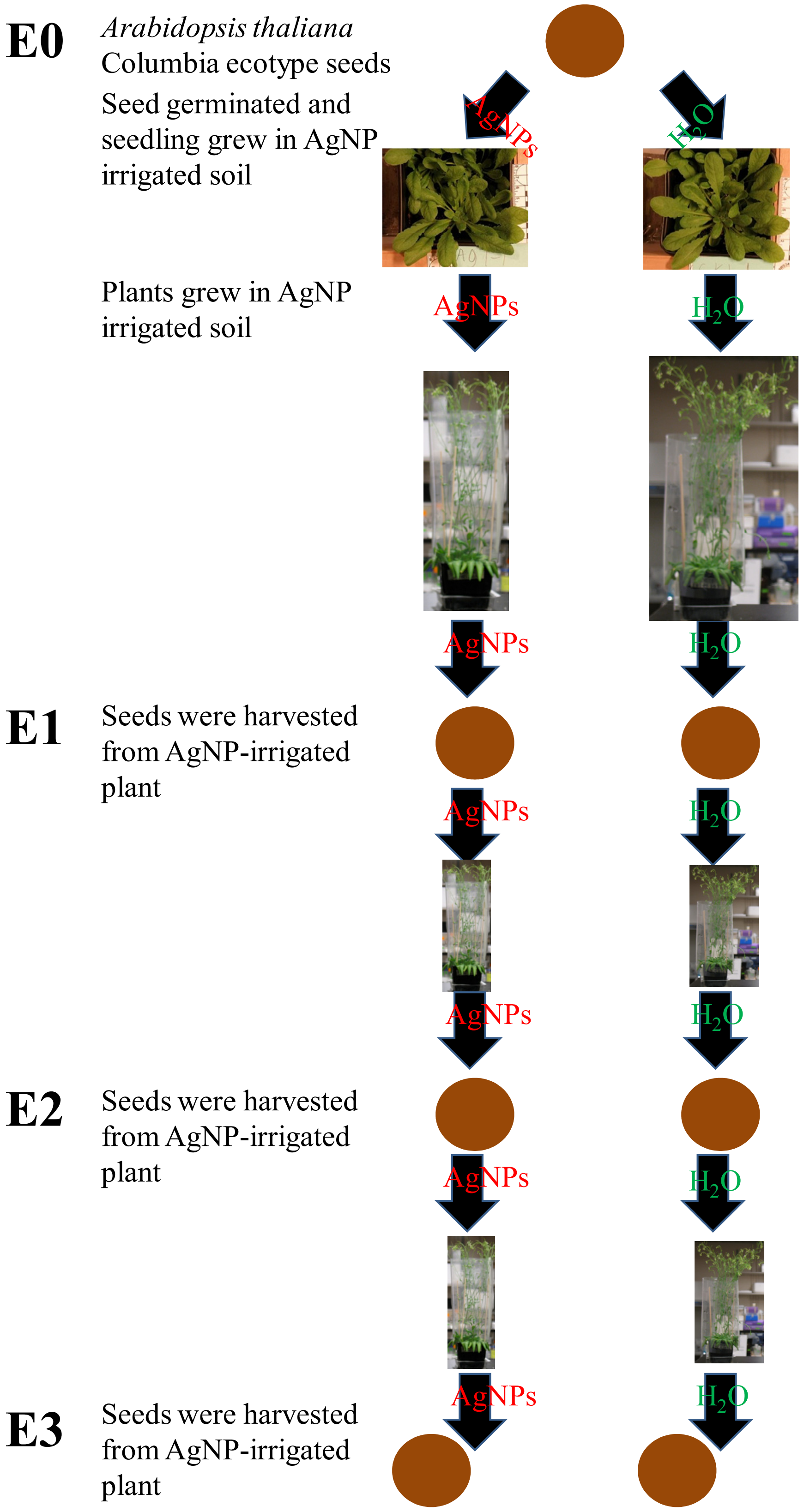

Figure S2. Reproductive traits of control, AgNP-treated and $AgNO_3$-treated plants. (**A**) Inflorescence height from 50 to 61 DAP; (**B**) Flower numbers from 52 to 64 DAP; (**C**) Siliques numbers from 57 to 64 DAP; (**D**) Seed weight (in mg) per 100 seeds for control, two concentrations of AgNPs 75 µg/L (as 75 AgNPs) and 300 µg/L (as 300 AgNPs) and two concentrations of $AgNO_3$ 4.25 µg/L (as 4.25 $AgNO_3$) and 17 µg/L (as 17 $AgNO_3$).

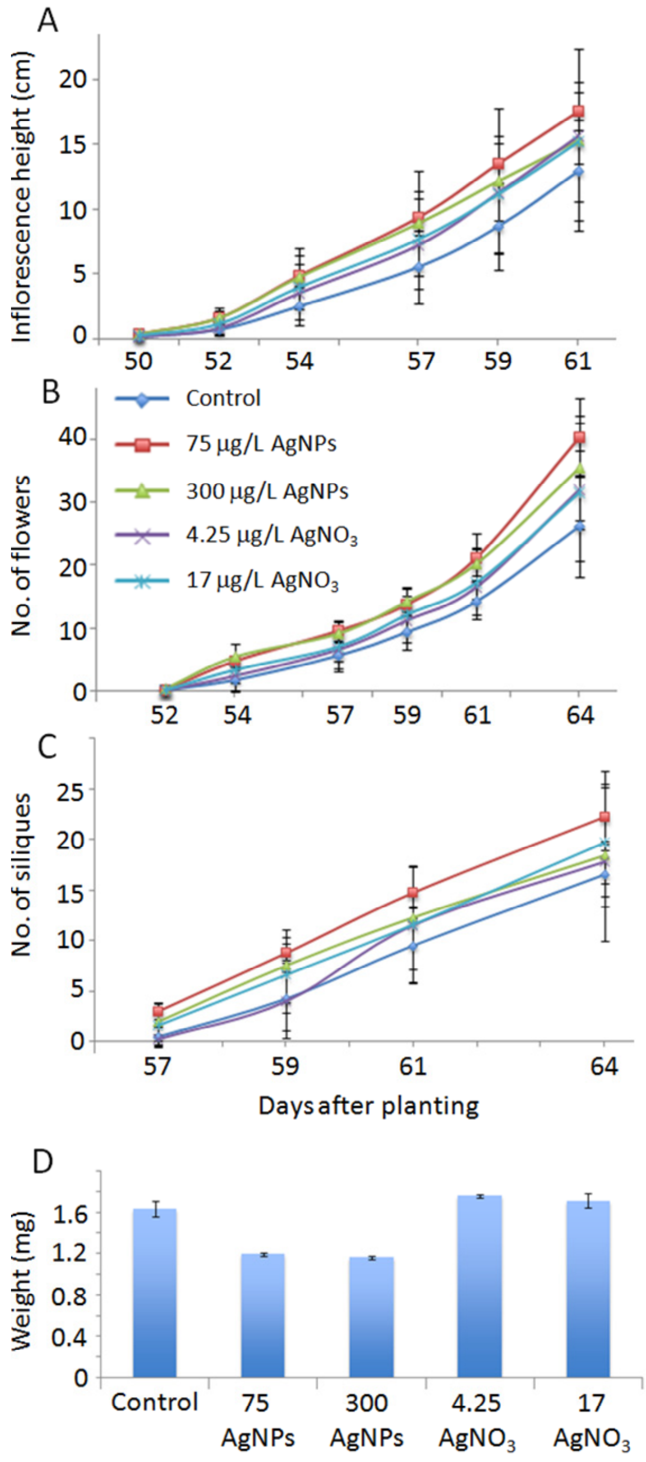

Figure S3. Accumulation of AgNPs in the Arabidopsis root tip at 14 DAP. (**A**) control plant; (**B**) AgNPs treated plant. Each figure (**A**,**B**) contains four panels: top left, AgNPs (in red); top right, GFP (in green); bottom left, brightfield; bottom right, composite of the three.

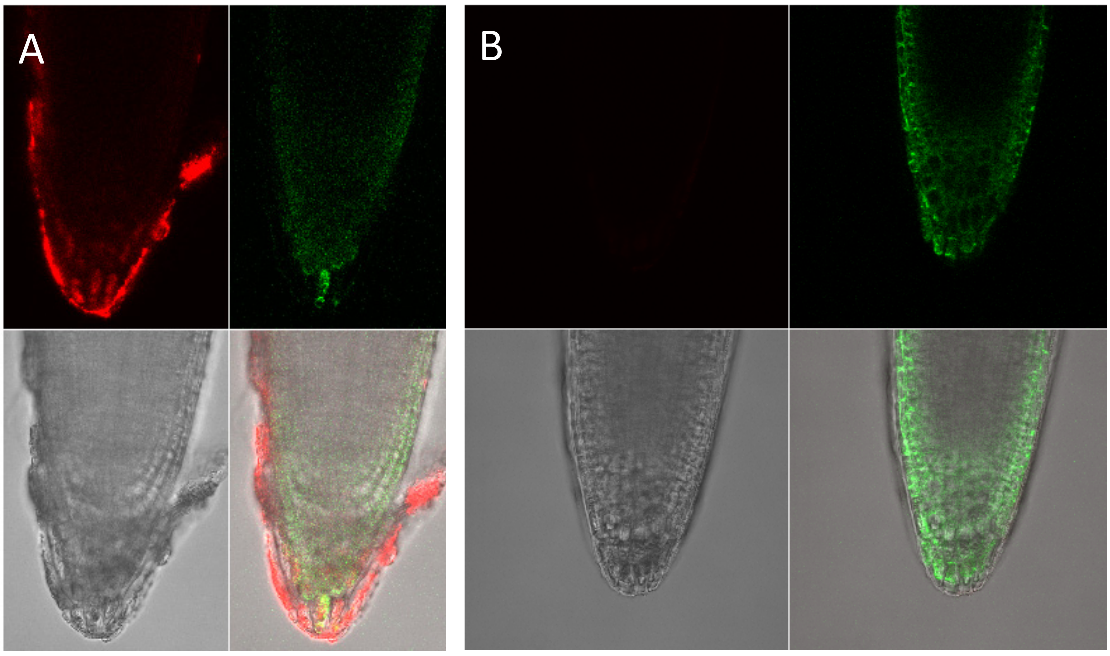

Figure S4. Original four panels for each of (**A**–**F**) in Figure 3. Four panels were: top left, AgNPs (in red); top right, GFP (in green); bottom left, brightfield; bottom right, composite of the three.

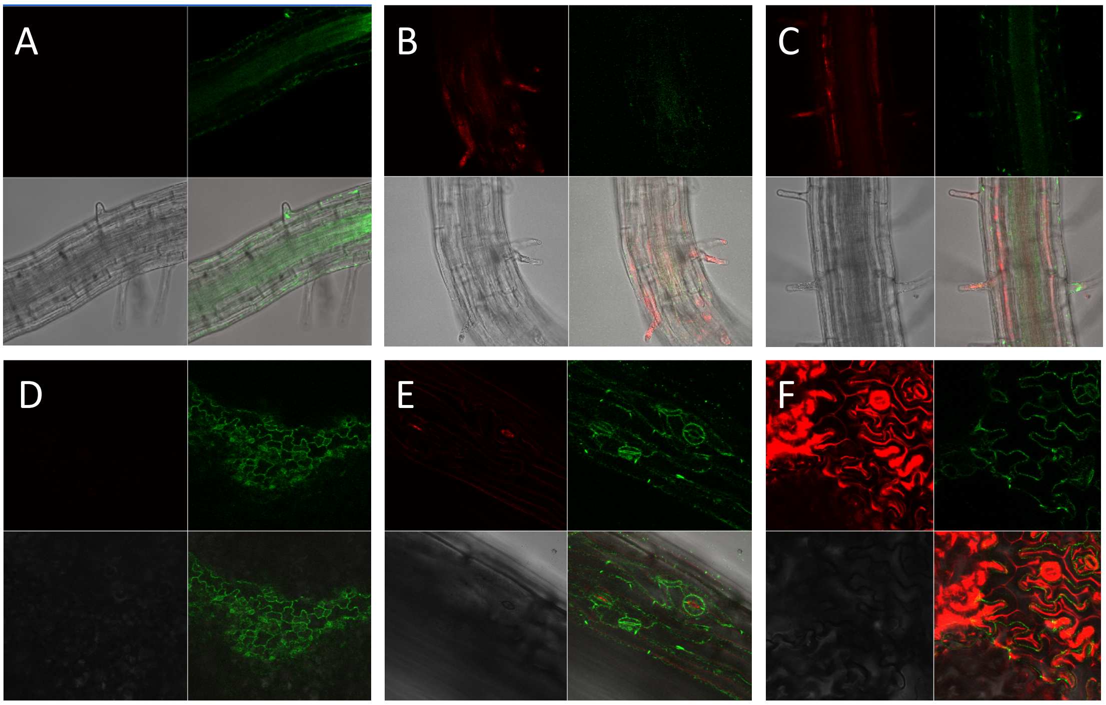

Figure S5. Using an energy dispersive X-ray spectroscopy (EDS) to view signals of Ag. (**A**) SEM image of one AgNPs-treated root hair; (**B**) EDS-detection of Ag (*i.e.*, turquoise color) in addition to show other different elements (C, O, K, S).

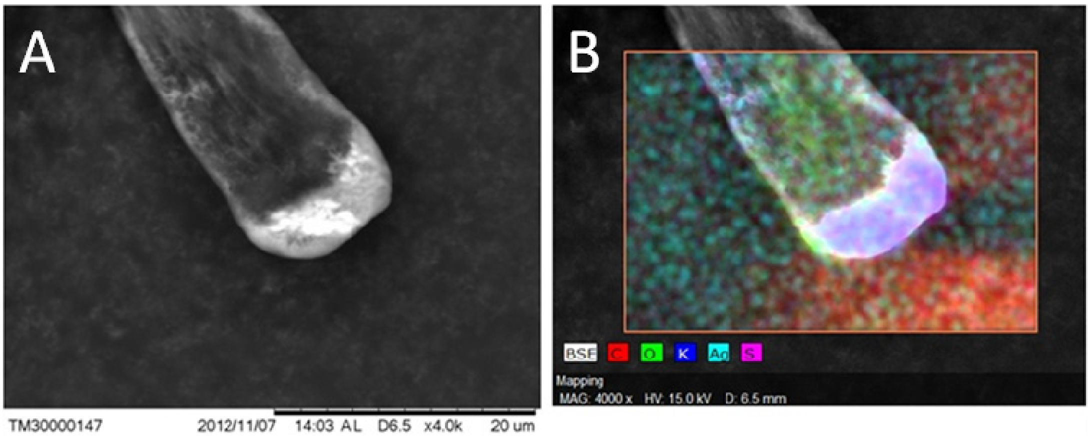

Figure S6. Lateral root primordia and lateral roots of AgNP-treated Arabidopsis plants. (**A**,**B**) 14 DAP; (**C**) 17 DAP; (**D**) control of 17 DAP. Each figure (**A**–**D**) contains four panels: top left, AgNPs (in red); top right, GFP (in green); bottom left, brightfield; bottom right, composite of the three. (**A**) Few AgNPs entered a lateral root, but overall the lateral root was functional. (**B**) Some AgNPs entered a lateral root primordium. (**C**) AgNPs already entered vascular tissue and its lateral root. (**D**) Control root does not have AgNPs.

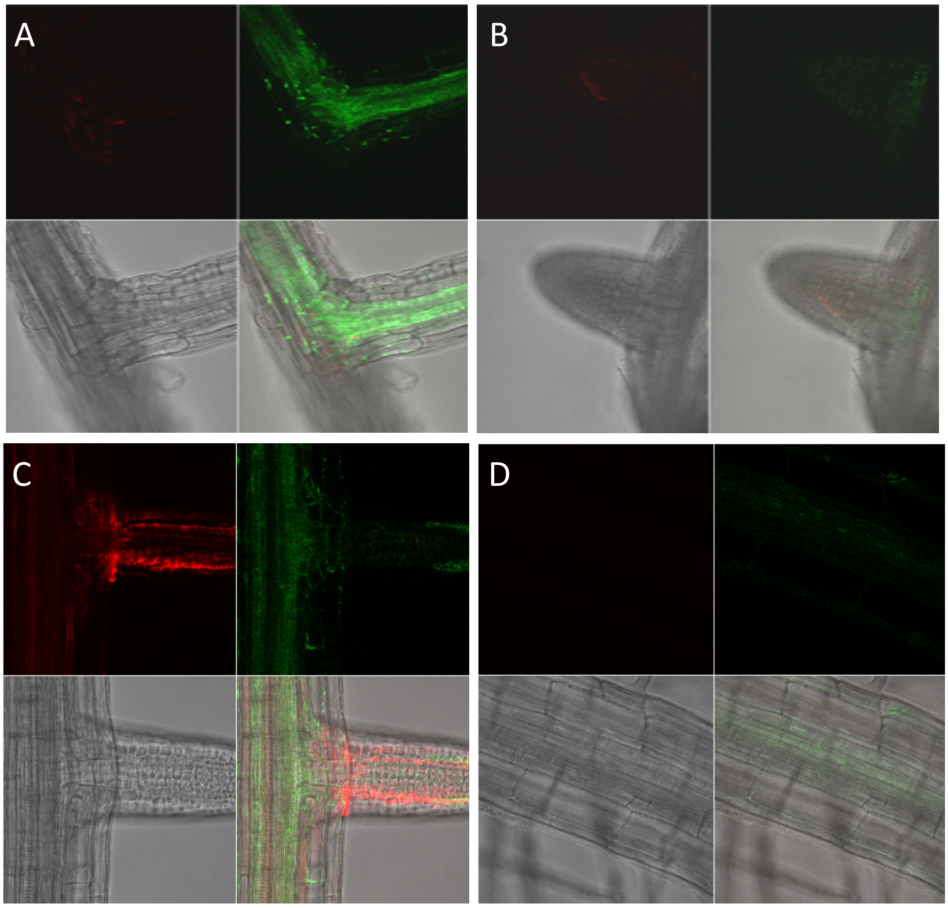

Figure S7. Detection of AgNP accumulation in root tips by SEM. (**A**) Overview of an Arabidopsis root with AgNP accumulation in its tip (black arrow); (**B**) Enlarged tip of (**A**).

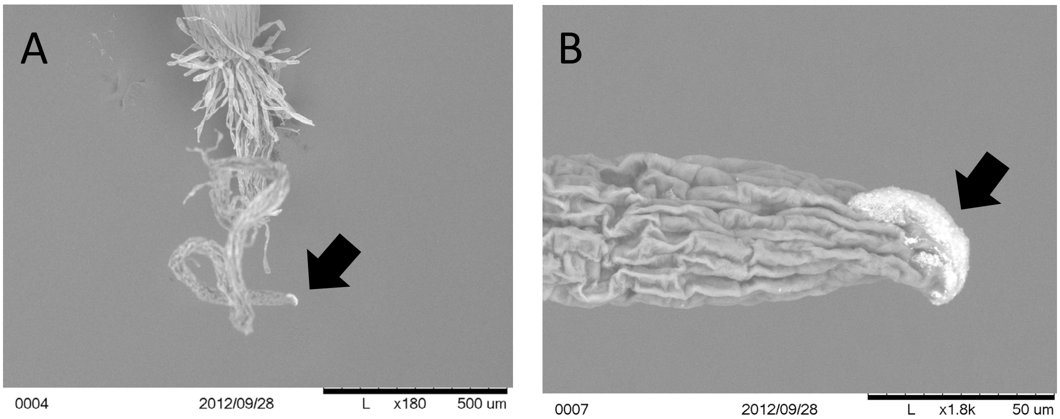

© 2014 by the authors; licensee MDPI, Basel, Switzerland. This article is an open access article distributed under the terms and conditions of the Creative Commons Attribution license (http://creativecommons.org/licenses/by/3.0/).
